# The Role of Lysine Methyltransferase SET7/9 in Proliferation and Cell Stress Response

**DOI:** 10.3390/life12030362

**Published:** 2022-03-02

**Authors:** Alexandra Daks, Elena Vasileva, Olga Fedorova, Oleg Shuvalov, Nickolai A. Barlev

**Affiliations:** 1 Institute of Cytology RAS, 194064 St. Petersburg, Russia; alexandra.daks@gmail.com (A.D.); slkd-k@mail.ru (E.V.); fedorovaolga0402@gmail.com (O.F.); oleg8988@mail.ru (O.S.); 2 Children’s Hospital Los Angeles, University of Southern California, Los Angeles, CA 90027, USA

**Keywords:** SET7/9, SETD7, lysine-specific methyltransferase (PKMT), cell proliferation, stress response, post-translational protein modification

## Abstract

Lysine-specific methyltransferase 7 (KMT7) SET7/9, aka Set7, Set9, or SetD7, or KMT5 was discovered 20 years ago, yet its biological role remains rather enigmatic. In this review, we analyze the particularities of SET7/9 enzymatic activity and substrate specificity with respect to its biological importance, mostly focusing on its two well-characterized biological functions: cellular proliferation and stress response.

## 1. Introduction

Methyltransferases are a compendium of diverse enzymes, most of which use S-adenosyl-methionine (Ado-Met) as a donor of methyl groups. The basic methyl group transfer reaction is the catalytic attack of a nucleophile (carbon, oxygen, nitrogen, or sulfur) on a methyl group to form methylated derivatives of proteins, lipids, polysaccharides, nucleic acids, and various small molecules. This methyl conjugation not only affects the bioconversion pathways of many drugs, but also affects the properties of endogenous neurotransmitters and hormones. Furthermore, methylation is fundamental to the regulation of gene expression. Unlike DNA methylation, which has been known since the middle of the last century, protein methylation was discovered relatively recently. Proteins can be methylated at different amino acids, however, for protein-protein interactions the most relevant and well-studied is methylation on lysine and arginine residues. Gene expression can be regulated by lysine methylation on two levels: methylation of histones and methylation of non-histone proteins that include transcription factors and chromatin modifiers.

## 2. The History of SET7/9 Discovery

SET7/9, a lysine methyltransferase (PKMT) encoded by the SETD7 gene ((su(var)3–9, enhancer of zeste, trithorax (SET) domain-containing protein 7) was discovered independently by two laboratories in 2001. Reinberg’s lab named this enzyme Set9 and Ye Zhang’s lab called it Set7 [1,2]. Later, these two names were unified as SET7/9. Studies from both groups identified SET7/9 as specific lysine 4 (K4) of histone H3 (H3K4) methyltransferase. Zhang’s group indicated that Set7 was able to di-methylate H3K4, which led to transcriptional activation by counteracting SuVar39h1-mediated H3K9 methylation. However, the caveat in the interpretation of the in vitro methylation data was that these experiments were done on free histones, whereas it is well known that the basic unit of chromatin is the nucleosome that is formed by a histone octamer wrapped by 157 nucleotides of DNA. Moreover, the in vivo experiments relied on the modification-specific antibodies, which are notoriously famous for their off-target recognition. Numerous experiments from different groups, including ours, have clearly demonstrated that SET7/9 failed to methylate nucleosomal histones. Furthermore, despite the early report, SET7/9 was convincingly shown to exert its functions as mono-methyltransferase but not as dimethyltransferase. For example, Dhayalan et al. showed that although SET7/9 was able to transfer two methyl groups to both histone- and non-histone targets in vitro, it did so with much lower efficacy (~10% of the mono-methylation rate) [3]. Extensive structural studies showed that the free-energy barrier for the transfer of the first methyl group by SET7/9 was 17–18 kcal/mol and the subsequent addition of the second methyl group imposed a 5 kcal/mol higher energy barrier for the transfer. Therefore, at least in vitro, SET7/9 acts preferentially as a monomethyltransferase. However, it should be noted that in cellulo studies in islet cells from R.G. Mirmira’s group suggested that SET7/9 was associated with the di-methylation of H3K4 [4]. One can speculate that SET7/9-mediated mono-methylation can trigger the subsequent addition of a second or third methyl group by other yet unknown methyltransferases [3,5,6].

## 3. The Substrate Specificity of SET7/9

Another enigmatic and debatable feature of SET7/9 is its substrate recognition specificity. There is an obvious discrepancy between the predicted frequency of occurrence for the potential SET7/9 consensus motif to be found in its target proteins and the handful number of in vivo confirmed substrates of SET7/9 that have been reported to date. Using the sequence-based approach together with the comparison of the structures of SET7/9 bound to TAF10, histone H3, and p53, a conserved sequence K/R-S/T/A-K*-D/N/Q/K (K* is the methylation site) was identified [7,8]. Later, it was clarified that the requirement for amino acid residues in +1 and +2 positions is not that stringent for the ability of SET7/9 to methylate the substrate. Thus, the majority of SET7/9 methylation targets share the G/R/H/K/P/S/T-K>R-S>K/Y/A/R/T/P/N-K* sequence motif [3]. However, in another study alternative, SET7/9-recognition amino acid sequences were reported. The p300/CBP-associated factor (PCAF), which is an acetyltransferase itself, was shown to be mono-methylated by SET7/9 methyltransferase at K78 and K89 in vitro forming the consensus motif A/F/I/V-K*-D/K (K* is the methylation site) for SET7/9 modification [9]. Comparison of 45 known methylation consensus motifs of SET7/9 shows high complexity of the target sequence. There are two types of consensus sequences that could be identified as preferable sites for SET7/9 methylation. Most analyzed proteins contain the K/R-S/A-K-K/S/R (Type 1) consensus motif and display an enrichment in the positively charged amino acids situated in the flanking regions, while the Type 2 alternative consensus sequence is enriched in basic amino acids in the flanking region and often displays proline at −12 position [10]. Our unpublished results indicate that the recognition sequence motif is much longer than it is thought currently (Vasileva, Daks and Barlev, unpublished).

## 4. Cellular Localization of SET7/9

Another interesting feature of SET7/9 is that it can be found preferentially either in the nucleus or in the cytoplasm, depending on the cell line [6,11,12,13,14,15]. Notably, unlike other SET domain-containing KMTs, SET7/9 does not contain a defined nuclear localization signal in its sequence. Thus, it can be hypothesized that SET7/9 is imported into the nucleus via a direct interaction with importin 5a, or via protein-protein interactions with its target proteins. This hypothesis is supported by the observation that nuclear factor (NF)-kappa-B (NFκB) recruits SET7/9 to the promoters of NFκB-dependent genes [11]. In light of the fact that NFκB interacts with the actin-binding protein, ACTN4, it would be interesting to see whether SET7/9 also interacts with elements of the cytoskeleton [16]. The SET7/9 localization may also depend on the cell type. For example, in mouse embryonic fibroblasts (MEFs), SET7/9 retains Yes-associated protein (YAP) in the cytoplasm [17]. In contrast, in human monocytes, SET7/9 was observed with NFκB-p65, both in the cytoplasm and nucleus [11]. Likewise, in the human osteosarcoma cell line, U2-OS, SET7/9 was detected both in the cytoplasm and the nucleus. However, upon DNA damage SET7/9 accumulates in the nucleus [18]. Therefore, further research is required to elucidate how SET7/9 is transported into the nucleus.

## 5. The Structural Organization of SET7/9 Methyltransferase

**Figure 1:**
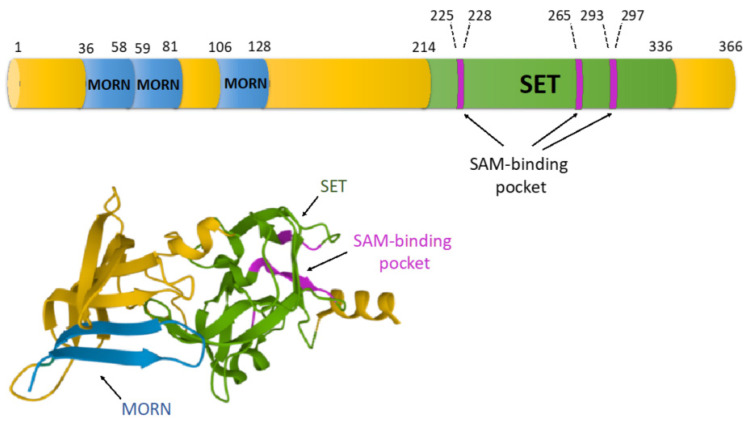
The domain organization and 3D-structure (PDB ID 1N6C, [26]) of the SET7/9 protein. The amino-terminal domain includes three MORN repeats. The SET domain is located in the C-terminus. Several amino acid residues of the SET domain form the SAM-binding pocket. SAM—S-adenosyl-L-methionine.

SET7/9 is a member of the SET domain-containing methyltransferases family that transfers a methyl group on the target protein involving S-adenosyl-L-methionine (SAM) as a donor. The protein structure of SET7/9 includes three MORN (Membrane Occupation and Recognition Nexus) domains mediating protein-protein interactions with the substrates and one SET domain required for SET7/9 enzymatic activity (Figure 1). According to the study of H. Liu et al. the MORN domain repeats represent a concave structure which is enriched in negatively charged amino acids [19]. Thus, perhaps expectedly, it can bind a number of positively charged proteins, including the DNA binding domains of several transcription factors. Using the bioinformatic approach, it was revealed that the MORN repeat-containing proteins are expressed both in procaryotes and eucaryotes [20]. In addition to SET7/9, there is a number of MORN repeat-containing proteins including junctophilins (JPHs), ALS2 (Rho guanine nucleotide exchange factor, alsin), and MORN4 (retinophilin) that are expressed in mammals [21,22,23]. It is generally assumed that MORN repeats bind to lipids and are responsible for plasma membrane targeting. However, there is a growing volume of evidence suggesting that MORN repeats may mediate protein-protein interactions [24,25]. Indeed, by using a GST-pull-down assay coupled with mass spectrometry, we have demonstrated that MORN repeats are responsible for the majority of SET7/9 interactions [10].

The resolved crystal structure of the SET domain revealed that the amino-terminal domain has a groove running across the extended beta sheet to the SET domain leading to a narrower channel running around the SET domain [27]. It was shown that this N-domain provides part of the binding site for basic histone tails as well as participates in determining the substrate specificity of the enzyme, while the C-terminus of the SET domain is important for the catalytic competence and contributes to the formation of the active site [27] (Figure 1).

## 6. Histone Targets of SET7/9

Posttranslational histone modifications such as methylation, phosphorylation, acetylation, ubiquitination and ADP-ribosylation define chromatin’s dynamic structure and function. Histones as substrates for lysine methylation were first described in 1964 [28]. In particular, lysines H3K4, H3K9, H3K27, H3K36 and H4K20 are the preferred sites for methylation [28,29]. SET7/9 was initially identified as a methyltransferase that methylates H3K4 facilitating transcriptional activation by displacing the histone deacetylase NuRD complex (HDAC) [1], (Table 1). Since SET7/9-mediated methylation of H3K4 enhanced the following acetylation of histones and the latter correlates with gene activation, it was implied that H3K4 methylation by SET7/9 should positively regulate transcription. The Reinberg’s group also demonstrated that the interplay between the Set9 and Suv39H1 histone methyltransferases was specific, as the methylation of H3K9 by another histone methyltransferase, G9a, was not affected by the Set9-mediated methylation of H3K4. Moreover, methylation of H3K4 was shown to reduce Suv39H1-mediated methylation at K9 of H3 histone (H3K9) [1]. In line with this notion is the fact that methylated H3K9 was shown to localize to a 20-kb silent heterochromatic region, whereas methylated H3K4 was detected exclusively in surrounding euchromatic regions [30].

Recently, it was shown that SET7/9 specifically methylates histone H1.4 at the K121, K129, K159, K171, K177 and K192 positions, competing for binding with the H3 histone protein. Methylation of H1.4 by SET7/9 upon binding to DNA tended to form less α-helix but more β-structure than unmethylated H1.4. There are two sites in H1.4 for methylation in vivo: K129 in the C-terminal domain and at K34 in the N-terminal domain. Methylation of H1.4 at K34 results in the reduction of the levels of acetylation by competition, contributing to the establishment of the proper heterochromatin patterns during differentiation [31].

Moreover, such modification as ADP-ribosylation of H3 by ARTD1 (PARP1) prevents H3 methylation by SET7/9, while poly(ADP-ribosyl)ation (PARylation) of histone H3 allowed subsequent methylation of H1 by SET7/9 [50]. Taken together, histone lysine methylation is a mark involved in the maintenance of genome expression and is dynamically regulated during the transcriptional activation.

In addition to histone H3 and H1, SET7/9 was also reported to methylate the free histones H2A and H2B [3,15]. Again, similar to H3, these histones were subject to SET7/9-mediated methylation only in a free state, and not as part of the nucleosomal core. The functional significance of these modifications is still unknown [3,15].

## 7. Non-Histone Targets of SET7/9

According to the PPI database, SET7/9 interacts with more than 120 different proteins, (BioGRID) and at present, more than 30 proteins are shown to be the targets of SET7/9. SET7/9 acts as regulator of such proteins as p53 [7], TAF10 [35], NFkB [43], YAP1 [17], PCAF [9], STAT3 [37], the nuclear receptors AR [51] and ERα [52], pRB [41] and many more. Perhaps it is not surprising that by regulating such crucial transcription factors SET7/9 participates in the orchestration of the cellular processes they are involved in. Here we focus on the effect of SET7/9 on cellular proliferation and stress response via methylation of the responsible factors.

### General Effects of SET7/9 on Transcription

Since lysine methylation on H3K4 is commonly associated with transcriptional activation, while H3K4me1 signatures are closely connected with the location and activity of multiple enhancers, it is tempting to speculate that SET7/9 plays role in tissue-specific transcriptional regulation [53]. Surprisingly, RNAi-mediated SET7/9 knockdown as well as somatic SET7/9 knockout do not affect global nucleosomal H3K4me in vivo [54], while in another report SET7/9 knockdown of rat mesangial cells led to the global H3K4me1 depletion [55]. This discrepancy requires further experimental validation.

A large volume of experimental data published to date unequivocally points to SET7/9 as a transcriptional regulator. In addition to free histones, whose fate and biological significance remains to be addressed in the future, SET7/9 also methylates basal transcription factors, e.g., TAF10 and TAF7. SET7/9 mono-methylates the TBP-associated factor TAF10, a component of the general transcription factor complex TFIID at a single lysine residue located at the loop 2 region, thereby increasing the affinity for RNA polymerase II. SET7/9-mediated methylation of TAF10 enhances transcription of several TAF10-dependent genes [35]. The in vitro studies also showed that SET7/9 is able to methylate TAF7 at the lysine residue K5 [8], which points to SET7/9 being involved in the TAF7-dependent regulation of its target genes, particularly in response to heat shock [56] (Table 1). 

Importantly, SET7/9 was also shown as a specific methyltransferase for Suv39H1, which methylates the latter at lysines 105 and 123. The SET7/9-methylated methylation of Suv39H1 results in heterochromatin relaxation and genome instability in response to DNA damage in cancer cells [32] (Table 1). 

In addition to histone methylation, SET7/9 can modulate gene expression by methylating DNA methyltransferase (DNMT1). The knockdown of SET7/9 was shown to stabilize cellular DNMT1 levels in mammalian cells, while the overexpression of SET7/9 decreased the DNMT1 protein level. The methylation-promoted degradation of DNMT1 facilitated DNA demethylation resulting in the approximately 10% reduction of global DNA methylation [33]. There is interplay between monomethylation of DNMT1 lysine at position 142 by SET7/9 and phosphorylation of DNMT1 at Ser143 by AKT1 kinase. In mammalian cells, phosphorylated DNMT1 is more stable than methylated DNMT1 [57]. Depletion of AKT1 increased methylation of DNMT1, thereby attenuating the DNMT1 level in cells. Thus, it is prudent to say that SET7/9is a regulator of DNMT1. However, given the low abundancy of DNMT1 methylation in vivo, additional research is required to establish the role of SET7/9 in the regulation of DNMT1.

## 8. SET7/9 and Cell Proliferation

### 8.1. SET7/9, β-Catenin and YAP1

**Figure 2:**
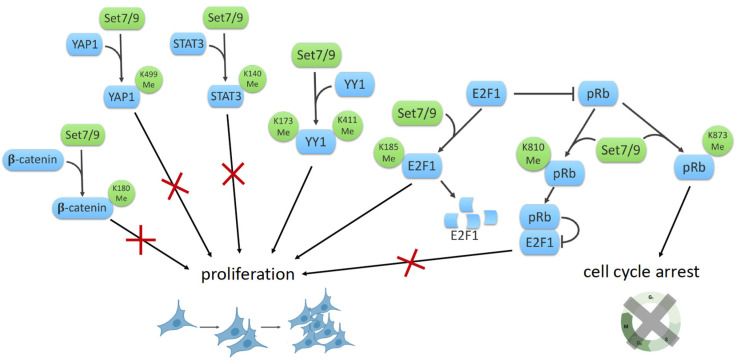
The scheme illustrating the participation of SET7/9 in regulation of proliferation.

β-catenin is a key mediator of the Wnt/β-catenin signaling pathway and plays an important role in cell fate determination, cell proliferation and tumorigenesis. SET7/9 was shown to monomethylate β-catenin at lysine residue 180 in vivo and in vitro, thereby providing a novel mechanism by which the Wnt/β-catenin signaling pathway is regulated in response to oxidative stress. The binding of Wnt to its receptor LRP5/6 induces dissociation of β-catenin and its negative regulator glycogen synthase kinase (GSK)-3β, resulting in the stabilization of the β-catenin protein, its translocation to the nucleus and subsequent transactivation of the target genes [36]. It was demonstrated that methylation of β-catenin by SET7/9 facilitates its phosphorylation by (GSK)-3β and subsequent β-catenin degradation. Expression of the Wnt/β-catenin target genes such as c-Myc and CyclinD1 were significantly enhanced by either the depletion of SET7/9 or the mutation in the methylation site (K180R) of the β-catenin protein to promote the growth of cancer cells [36] (Figure 2). 

On the other hand, SET7/9 was reported as a regulator of the methylation-dependent checkpoint in the Hippo/YAP1/TAZ pathway. SET7/9 monomethylates the YAP1 protein leading to its cytoplasmic retention [17]. YAP1 and TAZ are integral components of the β-catenin destruction complex while the β-catenin/TCF4 complex binds enhancer elements of the YAP gene to drive YAP expression in colorectal cancer cells [58,59]. Taken together, these facts indicate that SET7/9 may be considered as one of the key regulators of the Wnt/β-catenin and Hippo signaling pathways.

### 8.2. SET7/9 and STAT3

Another example of SET7/9 involvement in the regulation of the cell cycle is presented by the SET7/9-mediated methylation of the STAT3 transcription factor, which results in harnessing the activity of the latter. STAT3 forms dimers through reciprocal phosphor-tyrosine–SH2 interactions after phosphorylation on tyrosine and serine residues in response to different cytokines and growth factors. This phosphorylated form of STAT3 binds to and activates the promoters of its target genes. STAT3 can be methylated at K140 by SET7/9 and demethylated by LSD1. Methylation of K140 decreases the steady-state level of activated STAT3 and hence the expression of many STAT3 target genes [37] (Figure 2). 

### 8.3. SET7/9 and YY1

YY1 (Yin Yang1) is a multifunctional zinc-finger transcription factor involved in a variety of biological processes such as DNA repair, apoptosis, cell proliferation, differentiation and development. SET7/9 methylates YY1 at K173 and K411 positions and enhances the DNA-binding activity of YY1 both in vitro and in cellulo at specific genomic loci in cultured cells. Functionally, SET7/9-mediated methylation of YY1 augments its transcriptional function and hence cell proliferation [42].

### 8.4. SET7/9 and E2F1/Rb1

The retinoblastoma protein (pRb) is a tumor suppressor protein playing an important role in regulating progression through the early stages of the cell cycle. pRb negatively regulates entry into the S-phase, thereby affecting the early cell cycle control [60]. The retinoblastoma protein interacts and blunts transcriptional activity of the E2F (E2 promoter-binding factor) family of transcription factors [61]. In addition to cell cycle control, pRb activity is associated with other types of cell fate, such as differentiation, senescence and apoptosis [62,63]. Munro et al. demonstrated that SET7/9 regulates the pRb tumor suppressor activity by methylating it at the K873 position [40]. SET7/9-mediated methylation of the C-terminal region of pRb facilitates the interaction between methylated pRb and the heterochromatin protein HP1, resulting in pRb-dependent transcriptional repression, cell cycle arrest, and differentiation [40].

It should be mentioned that the interplay between methylation and phosphorylation was observed for histone and non-histone proteins. In line with this, the phosphorylation of pRb required for the release of E2F1 and hence cell cycle progression was attenuated by the methylation of pRb at K810 by SET7/9 [41,62,63]. Apparently, SET7/9 locks pRb in a hypophosphorylated, growth-arresting state, thereby limiting the E2F target gene expression. Thus, cell cycle control could be regulated by the methylation/phosphorylation switch.

**Figure 3:**
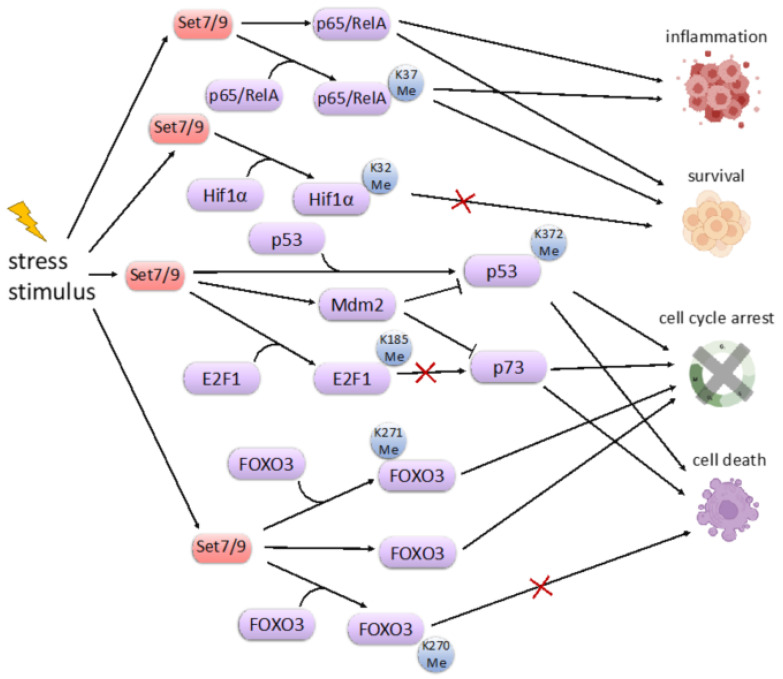
The scheme illustrating the key cellular stress effector pathways regulated by SET7/9. The functional outcomes are denoted on the right.

Moreover, the methylation of E2F1 by SET7/9 at lysine 185 inhibits acetylation and phosphorylation at the nearest positions, stimulating ubiquitination-depended degradation of the E2F1 protein [38]. At the same time, SET7/9 was shown as a critical co-activator of E2F1-dependent transcription under conditions of DNA damage [39]. SET7/9 affected the activity of E2F1 by indirect modulation of histone modifications in the promoters of E2F1-dependent genes, thereby promoting cell proliferation and repressing apoptosis. However, SET7/9 differentially affected E2F1 transcription targets: it promoted the expression of the CCNE1 gene, thereby facilitating cell proliferation, and it repressed the TP73 gene, hence preventing apoptosis [39] (Figure 3). Additionally, it was demonstrated that LSD1 removes the methyl mark required for the E2F1 stabilization and function in apoptosis [38]. Collectively, SET7/9 seems to be a critical element for the regulation of the cell cycle upon stress.

## 9. SET7/9 and Cell Stress Response

The tumor suppressor p53 was the first published non-histone methylation target for SET7/9. The p53 protein is the sequence-specific transcription factor that activates expression of its downstream transcription targets, whose products are involved in the regulation of cell cycle arrest and apoptosis [64]. The lysine-specific methylation of p53 at position K372 by SET7/9 is important for p53 transcriptional activation and stabilization mediated by its subsequent acetylation by p300/CBP [7]. Therefore, the cross talk between lysine methylation and acetylation is critical for p53 activation in response to DNA damage [18]. Importantly, both methylation and acetylation prevent p53 poly-ubiquitination mediated by an E3 ligase, Mdm2 [65]. The physical interaction between p53 and Mdm2 is critical for p53 ubiquitination and its subsequent degradation. Thus, small molecules that break this interaction were shown to stabilize p53 [66,67,68]. In line with this was the observation that SET7/9 physically interacts with Mdm2 and sequesters it away from p53 [69] (Figure 3). Both in vitro and in vivo experiments suggest that SET7/9 and Mdm2 have inverse expression. Accordingly, the unleashed expression of Mdm2 in cancer patients with diminished expression of SET7/9 correlated with poor survival outcomes [69].

It is worthy of note that SET7/9 was shown to be a new regulator of another p53-specific enzyme, Sirtulin 1 (SIRT1). The latter plays an important role during aging, metabolism and autophagy. SIRT1 interacts with SET7/9 mostly in response to DNA damage in human cells resulting in the dissociation of SIRT1 from p53 and the enhancement of p53 acetylation at K382. SET7/9 is able to both interact with and methylate SIRT1 at multiple sites [49]. The presence of SET7/9 attenuates the interaction between SIRT1 and p53, resulting in the transcriptional activation of p53 target genes and thereby inflicting cell cycle arrest and apoptosis [49].

The transcription factor FoxO3 of the Forkhead Box O (FoxO) family is involved in the regulation of the cellular response to ROS-induced DNA damage [70]. In response to oxidative stress, FoxO3 induces cell cycle arrest, apoptosis, autophagy, and hence can be considered as a tumor suppressor. It also affects metabolism and aging [46,70,71]. The phenotypic features of FoxO3-knockout mice support its involvement in the process of ageing and are exemplified by premature follicular activation, ovarian failure and early infertility [72].

FoxO3 inhibits transcription induced by ERα, thereby inhibiting the proliferation of breast cancer cells [73]. Accordingly, downregulation of the ERα activity is associated with poor prognosis in estrogen-dependent breast cancer and colorectal cancers [74]. SET7/9 methylates the FoxO3 protein at K271 [45]. Paradoxically, the methylation of FoxO3 destabilizes the protein but enhances its transcriptional activity towards the activation of pro-apoptotic genes. This paradoxical effect is similar to the one observed in the case of SET7/9-mediated methylation of E2F1. It should also be noted that SET7/9 apparently can methylate the additional FoxO3 lysine residue, K270 [46] (Figure 3). Surprisingly, this methylation has an opposite effect on the transcriptional activity of FoxO3, thereby preventing it from activation of the pro-apoptotic gene BIM and, hence, preventing cell death.

Hypoxia-inducible factor 1α (HIF-1α) is another target of SET7/9 methylation activity. HIF1a is a critical transcription factor for cellular hypoxic response. In response to oxygen deprivation, HIF1a is released from the ubiquitin-mediated degradation mediated by the von Hippel-Lindau disease tumor suppressor (VHL) E3 ligase [75]. SET7/9 methylates HIF-1α at K32, which competes with ubiquitination and its subsequent degradation. Thus, SET7/9 stabilizes the HIF-1α protein and stimulates the HIF-1α-dependent transcription of genes involved in the regulation of energy metabolism and angiogenesis to maintain tissue homeostasis [48]. However, several studies suggest that SET7/9-mediated methylation inhibits HIF1α transcriptional activity by preventing its DNA binding (Figure 3). Importantly, this effect was reversed by a SET7/9-specific inhibitor, (R)-PFI-2 [47,48]. Thus, additional experiments are required to elucidate the effect of SET7/9 on the function of HIF-1α.

## 10. Concluding Remarks

To assess the biological significance of SET7/9’s role in proliferation and stress response in vivo, it is important to develop the relevant tools. In this respect, small-molecule inhibitors are promising tools that allow for the probing functions of methyltransferases in diseases. In this respect, Barsyte-Lovejoy et al. designed and synthesized a novel inhibitor, (R)-PFI-2, against SET7/9 [76]. This inhibitor has demonstrated low toxicity even at high concentrations in human cells [77]. Mori et al. has developed an inhibitor which is an amine analogue of adenosylmethionine, bearing various alkylamino groups for increasing the inhibitory activity [78].

Berberine is a naturally occurring isoquinoline alkaloid which is commonly used in traditional Chinese medicine and exhibits anti-oxidant, anti-inflammatory, and anti-cancer activities. Berberin was shown to augment the activity of SET7/9 towards NFκB by sensitizing human cancer cells to ionizing radiation or chemotherapy. Berberine negatively regulates NFκB through SET7/9-mediated lysine methylation. Such methylation leads to a decrease in miR-21 levels and Bcl-2 levels [79].

Since there is a significant correlation between SET7/9 and different types of cancer, the wide application of small molecules as experimental tools should significantly facilitate the experimental work directed towards the elucidation of SET7/9 in tumorigenesis and other diseases.

## Figures and Tables

**Table 1:** The substrates of SET7/9-dependent methylation playing roles in proliferation and cellular stress response.

SET7/9 Target Protein	Methylation Sites	Effect of the Modification	Reference
Histone H1	K12, K14, K17, K20, K21, K27, K111	Modulation of the affinity of histone H1 to chromatin during human pluripotent cells differentiation	[31]
Histone H1.4	K34, K127, K129, K130	Prevention of acetylation at the same sites, heterochromatin formation	[31]
Histone H2.A	K5, K13, K15	Unknown	[3]
Histone H2.B	K15	Unknown	[3]
Histone H3	K4	Activation of transcription	[1]
Suv39H1	K25, K123	Heterohromatin relaxation, genome instability	[32]
DNMT1	K142	Promotion of DNMT1 ubiquitination and proteasomal degradation	[33]
	K1094	Decrease of the DNMT1 level	[34]
TAF7	K5	Enhancement of TAF7 activity as co-factor of RNA polymerase II	[8]
TAF10	K189	Enhancement of TAF10 activity as co-factor of RNA polymerase II, activation of transcription of TAF10 target genes	[35]
YAP1	K494	Retention of YAP1 in the cytoplasm	[17]
β-catenin	K180	Promotion of β-catenin ubiquitination by (GSK)-3b and its subsequent proteasomal degradation	[36]
STAT3	K140	Dissociation of STAT3 from promoter elements, downregulation of STAT3-dependent genes expression	[37]
E2F1	K185	Promotion of E2F1 ubiquitination and subsequent proteasomal degradation	[38]
	Unknown	Enhancement of E2F transactivation of its target genes	[39]
pRb	K873	Enhancement of pRB-dependent repression of transcription	[40]
	K810	Promotion of p65/RelA ubiquitination and its subsequent proteasomal degradation	[41]
YY1	K173, K411	Retention of YY1 in the cytoplasm	[42]
p65/RelA	K37	Translocation to the nucleus and transactivation of target genes	[43]
	K314, K315	Promotion of p65/RelA ubiquitination and subsequent proteasomal degradation	[44]
FOXO3	K270	Downregulation of FOXO3-dependent transactivation of BIM	[45]
	K271	Increase of the FOXO3 transactivation potential	[46]
Hif1α	K32	Suppression of Hif1α transactivation of its target genes	[47,48]
p53	K372	Stabilization, translocation to the nucleus and transactivation of target genes	[7]
SIRT1	K233, K235, K236, K238	Enhancement of SIRT1-dependent p53 acetylation and activation	[49]
